# Current Status of Chikungunya in India

**DOI:** 10.3389/fmicb.2021.695173

**Published:** 2021-06-24

**Authors:** The Translational Research Consortia (TRC) for Chikungunya Virus in India, Anitha Jagadesh

**Affiliations:** Manipal Institute of Virology, Manipal Academy of Higher Education, Manipal, Udupi, Karnataka, India; Vector-Borne Diseases Group, International Centre for Genetic Engineering and Biotechnology (ICGEB), New Delhi, India; Infectious Disease Biology, Institute of Life Sciences, (Autonomous Institute of Department of Biotechnology, Government of India), Nalco Square, Bhubaneswar, India; Department of microbiology, All India Institute of Medical Sciences, (AIIMS), Bhubaneshwar, India; Department of Virology, Post-Graduate Institute of Medical Education and Research, Chandigarh, India; Department of Microbiology, TNMC and BYL, Nair Charitable Hospital, Mumbai, Maharashtra, India.

**Keywords:** Chikungunya fever (CHIKF), chikungunya virus (CHIKV), polyarthralgia, epidemiology, disease resolution

## Abstract

Chikungunya fever (CHIKF) is an arbovirus disease caused by chikungunya virus (CHIKV), an alphavirus of Togaviridae family. Transmission follows a human-mosquito-human cycle starting with a mosquito bite. Subsequently, symptoms develop after 2–6 days of incubation, including high fever and severe arthralgia. The disease is self-limiting and usually resolve within 2 weeks. However, chronic disease can last up to several years with persistent polyarthralgia. Overlapping symptoms and common vector with dengue and malaria present many challenges for diagnosis and treatment of this disease. CHIKF was reported in India in 1963 for the first time. After a period of quiescence lasting up to 32 years, CHIKV re-emerged in India in 2005. Currently, every part of the country has become endemic for the disease with outbreaks resulting in huge economic and productivity losses. Several mutations have been identified in circulating strains of the virus resulting in better adaptations or increased fitness in the vector(s), effective transmission, and disease severity. CHIKV evolution has been a significant driver of epidemics in India, hence, the need to focus on proper surveillance, and implementation of prevention and control measure in the country. Presently, there are no licensed vaccines or antivirals available; however, India has initiated several efforts in this direction including traditional medicines. In this review, we present the current status of CHIKF in India.

## Introduction

Chikungunya fever (CHIKF) is an arthropod-borne viral (arbovirus) disease (Mohan, 2006; Mohan and Sharma, 2007) that has become a global health concern following its resurgence since 2006 (Pialoux et al., 2007; Simon et al., 2008). Historically, there have been reports of epidemics of fever along with arthralgia as far back as 1824, similar to present day CHIKF (WHO, 2012; Puntasecca et al., 2021). However, the CHIKF was first officially described in 1952 after an outbreak on the Makonde Plateau, south-eastern Tanzania (Lumsden, 1955; Robinson, 1955). The word “chikungunya” is taken from the verb “kungunyala” in Kimakonde language spoken in the same area which means “to dry up or become contorted” (Lumsden, 1955; Benjamin, 2012). The disease develops the stooped posture due to the rheumatologic manifestations describing the word chikungunya. In Congo region it is known by the name “Buka Buka” meaning “broken-broken” representing incapacitating joint pain (Muyembe-Tamfum et al., 2003).

## Classification

**FIGURE 1:**
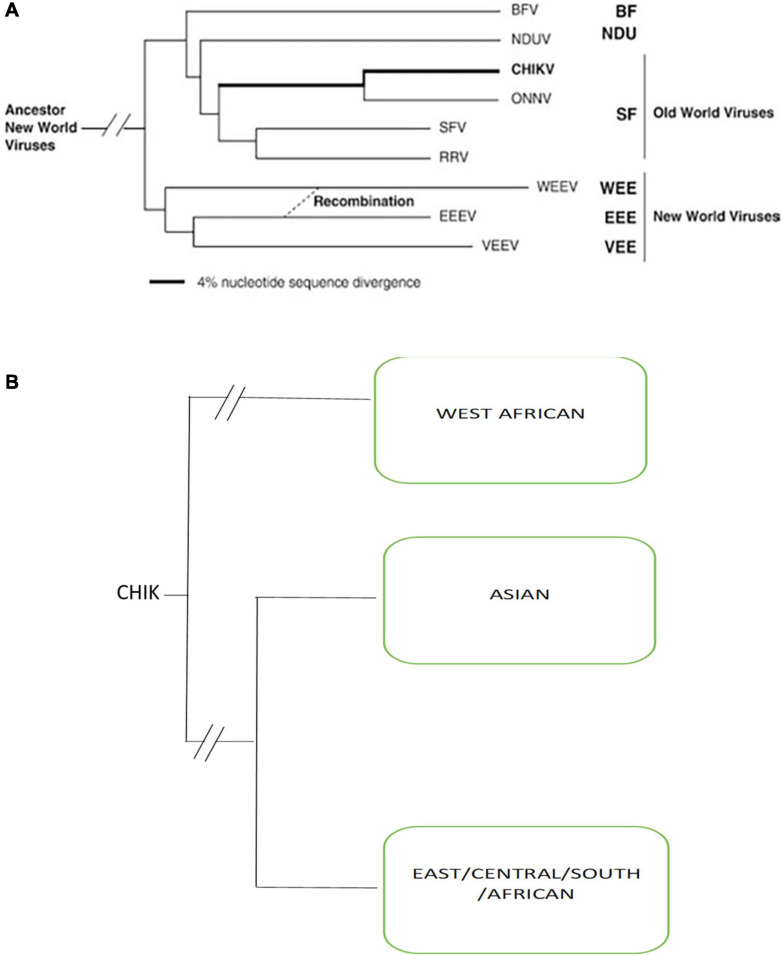
**(A)** Simplified Phylogenetic tree of all *Alphaviruses* generated from partial E1 envelope glycoprotein gene sequences by using the neighbor-joining program Adapted from Powers et al. (2001) (BF, Burma Forest virus; CHIKV, chikungunya virus; SF, Semliki Forest; WEE, western equine encephalitis; EEE, eastern equine encephalitis; VEE, Venezuelan equine encephalitis; NDUV, Ndumu virus; ONNV, o’nyong’nyong virus; RRV, Ross River virus). **(B)** Phylogenetic tree of three genotypes of chikungunya viruses.

Chikungunya virus (CHIKV) belongs to the genus Alphavirus within the family Togaviridae. Alphaviruses include all viruses of this family that are animal pathogens and have a worldwide geographic distribution based on which they are described classically as either Old World or New World viruses. The New World alphaviruses are distributed across the Americas and cause encephalitis in equines and humans. For example, the eastern equine encephalitis virus, western equine encephalitis virus, and Venezuelan equine encephalitis virus. Whereas Old World alphaviruses to which CHIKV belongs, cause fever, rash, and arthritis in humans, other members of the group include Sindbis virus, o’nyong’nyong virus, Ross River virus, Mayaro virus, Barmah Forest virus, and Semliki Forest virus are present in Asia, Europe, Australia, and parts of Africa (Figure 1). Most alphaviruses are transmitted by arthropod vectors that probably control their geographic dispersal and it is likely that several transoceanic exchanges might have occurred. The alphavirus CHIKV belongs to the Semliki forest virus complex of the *Alphaviruses*, as seen in Figure 1.

## Clinical Manifestations

After CHIKV infection most individuals develop symptoms after a silent incubation period that range from 2 to 6 days (can extend up to 12 days) followed by the onset of abrupt symptoms which include high fever and severe arthralgia. The clinical symptoms associated with CHIKV infection are classically divided into acute and chronic phases (Suhrbier, 2019). The acute stage is the febrile phase and can extend until 15 days since the onset of fever. This phase is characterized by common symptoms such as high fever and arthralgias; however, non-classical symptoms, tabulated in Table 1 may also occur during this phase. Fever is usually high and is poorly responsive to antipyretics. Anorexia, myalgias, nausea, and vomiting in adults, and even transient confusion in elderly patient are common with 50–97% of individuals developing these symptoms. Additionally, other less common symptoms like back pain, headache and fatigue have also been reported during this phase (Table 1). Bilateral polyarthralgia is noted as a typical symptom affecting mainly the small joints such as ankles, wrist and phalanges and few large joints such as knees and elbows (WHO, 2009). In about half of the cases, acute phase is also characterized by cutaneous lesions like maculopapular rashes, edematous or itchy skin affecting mainly the face and trunk (Simon et al., 2011). Symptoms such as diarrhoea, abdominal pain, nausea, and vomiting are seen in 15–47% of individuals (Thiberville et al., 2013). Other symptoms are asthenia, erythema, persistent conjunctivitis, conjunctival effusion, and cervical lymphadenopathy. Moderate thrombocytopenia, leukopenia and lymphopenia are also common (Staples et al., 2009; Staples and Fischer, 2014; Madariaga et al., 2016). Severe symptoms like bleeding due to thrombocytopenia, bulbous skin lesions, hepatitis, meningoencephalitis, meningitis have been reported in a few patients (Markoff, 2015; Weaver and Lecuit, 2015; Abeyratne et al., 2018; Saha et al., 2019). Ocular complications like retinitis or uveitis, myocarditis, nephritis, cranial nerve palsies and Guillain-Barre Syndrome have been observed for the first time during the 2006 Indian Ocean outbreak (Wielanek et al., 2007; Lebrun et al., 2009) as referred in Table 1. Renal and neurological complications are also associated with severe chikungunya infection (Weaver and Lecuit, 2015; Mercado et al., 2018). In few cases a very high viral load leads to persistence of the virus in minor joints during acute stage (Thiberville et al., 2013).

**TABLE 1:** Classical and non-classical clinical manifestations in chikungunya infection.

Clinical features of CHIKV infection	
Classical features	Complications
Fever	Bulbous skin lesions
Arthralgia	Fulminant hepatitis
Rashes on skin	Meningoencephalitis
Headache	Retinitis
Back pain	Uveitis
Nausea	Myocarditis
Vomiting	Nephritis
Joint swelling	Convulsions
Myalgia	Cranial nerve palsy
Lymphadenopathy	Guillain-Barre Syndrome
Fatigue	Acute renal failure
Restlessness	Respiratory failure
Anorexia	Meningitis
Abdominal pain	
Diarrhea	
Leukopenia	
Lymphopenia	

The disease is self-limiting and usually resolve within 2 weeks. However, about 30–40% infected patients can develop chronic stage which is defined as three months after the bulbous skin lesions onset of infection that can last up to several years with persistent polyarthralgia (Schwartz and Albert, 2010).

Chronic stage of the disease is associated with the development of inflammatory joints after the resolution of the acute phase and can last up to several years in some patients. The symptoms of this phase are mainly polyarthralgia and/or polyarthritis, mostly affecting small joints, such as phalanges and wrists, as well as large joints such as shoulders, knees, and ankles affecting the mobility of the affected patients (Hoarau et al., 2010). Inflammation of joints is seen for years, which progresses to chronic inflammatory rheumatism (Borgherini et al., 2008; Rajapakse et al., 2010; Simon et al., 2011; Gupta et al., 2014). Studies in other alphaviruses reveal that there is persistence of viral antigens which causes inflammation of joints (Suhrbier and Mahalingam, 2009; Suhrbier et al., 2012). In case of CHIKV, some studies have shown evidence of prolonged persistence of CHIKV RNA and viral proteins in the synovial tissues (Hoarau et al., 2010). Conversely, other studies have failed to observe viral particles in CHIKV infected joints during the chronic phase and argue that this phase is an outcome of host immune response to the infection (Chang et al., 2018). These contradictory findings suggest that more in-depth studies are required to understand the basis of disease progression to chronic phase in CHIKF.

## Clinical Diagnostic Matrix

**FIGURE 2:**
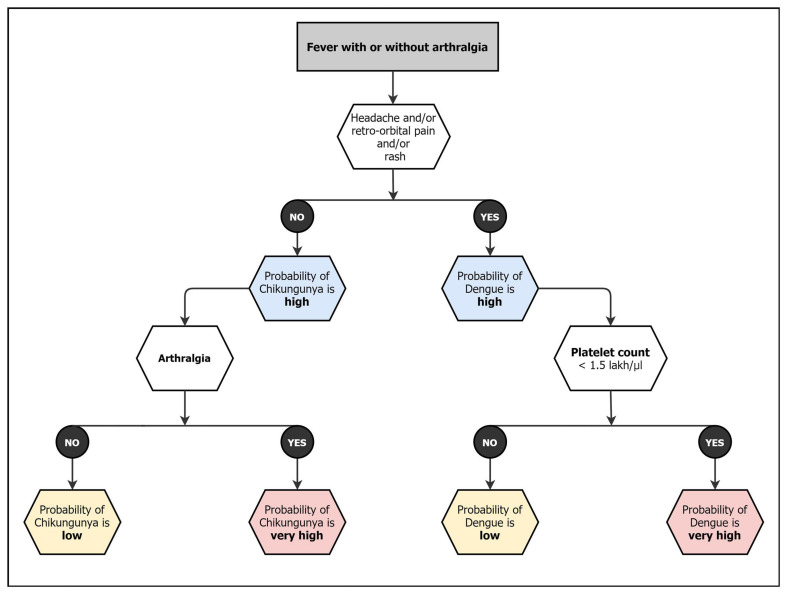
Diagnostic matrix followed by Indian clinicians for CHIKF and related coinfections (Lee et al., 2012).

In a country like India, which is an endemic area for chikungunya, Dengue, and malaria, the seasonality of these infections overlaps. Chikungunya and Dengue have a common vector and shows similar clinical presentation; hence coinfections are also reported in several studies (Saswat et al., 2015; Londhey et al., 2016; Jain et al., 2017; Kaur et al., 2017). Despite showing similar symptoms, the outcomes, and management strategies of these two viruses are vastly different. It is important for clinicians to make the differential diagnosis on the basis of various clinical presentations and laboratory methods to start appropriate treatment and avoid complication such as hemorrhages, Acute respiratory distress syndrome (ARDS), renal failure and arthritis (Kaur et al., 2018). It has been shown that simple clinical and laboratory variables can predict these infections at presentation and classify them into symptoms and laboratory tests for appropriate management (Lee et al., 2012). Acute arthritis, shorter duration of fever, rash, myalgia/arthralgia, and conjunctivitis are more prominent in CHIKF, while abdominal pain, leukopenia, neutropenia, and thrombocytopenia are more prominent in dengue fever (DF). Similarly, significant difference is noticed in platelet level cut-offs between DF and CHIKF along with bleeding in patient (Figure 2; Halstead et al., 1963; Nimmannitya et al., 1969; Hochedez et al., 2008; Kularatne et al., 2009; Laoprasopwattana et al., 2012; Lee et al., 2012; Thiberville et al., 2013; Taraphdar et al., 2015).

There is no specific antivirals or treatment regimen available for chikungunya and symptomatic treatment is the main stay of patient management. Generally, patient is advised for adequate rest and intake of plenty of water to prevent dehydration. Non-steroidal anti-inflammatory drugs are avoided until dengue infection is ruled out. Acetaminophen or paracetamol is given to reduce pain and fever (NVBDCP, 2014-15; CDC, 2018).

## Laboratory Diagnosis

In India, the choice of CHIKV test depends on various factors that include the intent of the test, resource availability and time of sample collection (days after onset of illness) (Figure 2). The diagnosis can be achieved by virus isolation, detecting antigen, nucleic acid, and IgM/IgG antibodies. Lab diagnosis is seriously limited by clinician not requesting a PCR test for chikungunya early in infection. The popular test is CHIK IgM, however, some tests show cross reactivity and give false positivity. Generally, the public hospitals receive National Institute of Virology CHIKV IgM kit. Each test has specific advantages, and disadvantages and combination of tests can be performed to increase the credibility of the test result. The laboratory diagnosis of CHIKV can be differentiated into two categories- classical and molecular diagnostics.

### Classical Diagnostics

Virus isolation was considered as the gold standard for CHIKV diagnosis for decades but is not commonly used in routine diagnosis now. Virus is detectable in the patient’s serum or plasma samples in high titers between 2 and 6 days following onset of illness. However, the viral load starts decreasing from day five after the onset of illness (Álvarez-Argüelles et al., 2019). Therefore, the usefulness of the test for diagnostic purpose is only limited to the acute phase of the infection. Viral growth and its identification are a time-consuming process and require specialized equipment and skilled staff for performing the procedure. However, the advantage of cell culture is that it allows amplification of virus and identification of viral strain that can be characterized for further epidemiological and research studies (Natrajan et al., 2019).

In the later stages of CHIKV infection (>5 days post-infection), the CHIKV IgM antibody capture ELISA is a dependable testing method for diagnosis (Álvarez-Argüelles et al., 2019). CHIKV specific IgG antibody ELISA can be used for sero-diagnosis by testing in a paired serum samples from the acute and convalescent phase of CHIKV infection; four-fold or more increase in the antibody titer should confirm the presence of recent CHIKV infection (Weaver and Lecuit, 2015). However, the collection of paired serum sample is often not possible, therefore, CHIKV specific IgM antibody in the acute-phase serum sample is generally used for diagnosis (Álvarez-Argüelles et al., 2019). Previous studies have demonstrated the persistence of CHIKV specific IgM antibody up to 10 months post-infection, Chelluboina et al. (2019) which proves that anti-CHIKV IgM antibody ELISA cannot be used as definitive diagnosis method. The cross-reactivity with other arboviruses like o’nyong’nyong virus and Mayaro virus is another disadvantage that affects the sensitivity of the assay (Prat et al., 2014).

Immunofluorescence assay (IFA) is a sensitive test that can be used for diagnosis of CHIKF, by detecting type-specific antibody against CHIKV. It is a labour-intensive method and requires skilled personnel to perform the procedure (Natrajan et al., 2019; Álvarez-Argüelles et al., 2019). This method is usually followed by plaque reduction neutralization test (PRNT) and micro neutralization test (MnT) that shows high sensitivity and specificity (Azami et al., 2016). However, it is not routinely used for diagnostic purpose as it is a tedious process (Álvarez-Argüelles et al., 2019).

### Molecular Diagnostics

Molecular diagnosis of acute phase CHIKV by RT-PCR is a commonly used method nowadays. It is a less time consuming and more specific technique. Serum and plasma are the preferred samples, but saliva and urine can also be used as an alternative and in the case of meningitis or encephalitis, CSF can be taken (Musso et al., 2016; Acevedo et al., 2017). Both Real-time RT-PCR and RT-PCR are commonly used and almost all genes of the virus are used for diagnostic purposes (Carletti et al., 2007; Edwards et al., 2007; Chen et al., 2013; Chiam et al., 2013; Parashar et al., 2015; Sarangan et al., 2018). Multiplex assays are also available for the simultaneous detection of CHIKV with other arboviruses (Mishra et al., 2011; Chen et al., 2015). Sensitivity of detecting chikungunya in commercial multiplex kit is a challenge and there is an urgent need to address molecular diagnostics of chikungunya in the country. In addition, commercial immunochromatography antigen detection kits are gaining popularity in the recent past owing to their high sensitivity and specificity with no cross-reactivity with other arboviruses (Okabayashi et al., 2015; Jain et al., 2018a). Other diagnostic initiatives are the use of biosensors using different technologies such as electrochemical impedance spectrometry and optical fibers that have shown promise in CHIKV diagnosis (George et al., 2019, 2021).

## Epidemiology of CHIK

Chikungunya outbreaks have been reported in the Indian subcontinent since the early 20th century. Since the earliest outbreaks, even though maximum outbreaks have been reported in India, sporadic have been reported in the neighboring countries. The following section describes the outbreaks that have occurred in the neighboring countries and in India. Summarizing the studies thus far, it appears that India may have been the epicenter of dispersal of CHIKV to neighboring as well as distant countries since the emergence of the IOL, which is significantly endemic to India (Newase et al., 2020).

### Epidemiology of CHIKV in Indian Subcontinent and Southeast Asia

As far as neighboring countries are concerned, CHIKV infection has been reported in multiple regions through the period of 2005–2017. During the period 2005–2006 outbreak in South Asia, particularly Myanmar and Sri Lanka, CHIKV positive cases were around 4.6 and 6.1%, of the total suspected cases, respectively; and in Southeast Asia, particularly Indonesia, the Philippines and Vietnam, the same were 27.4, 26.8, and 25.0%, respectively (Ngwe Tun et al., 2016). The Indian Ocean Lineage (IOL), within the ECSA genotype was associated with 2005–2006 outbreak in the Indian subcontinent and the Indian Ocean Islands. The E1-A226V mutation was associated with the reemergence which increased the adaptability of CHIKV to *Aedes albopictus* mosquitoes (Tsetsarkin et al., 2007) and it has been reported that this species of mosquitoes is predominant in Indian state of Kerala as well as the neighboring island nation, Sri-Lanka (Kumar et al., 2008; Hapuarachchi et al., 2010). In 2006–2007, General Hospital in Peradeniya, Sri Lanka reported an outbreak which was later confirmed as CHIKV infection. Moreover, Bangladesh has had a share of recurrent CHIKV infections during 2008, 2011, and 2017 (Khatun et al., 2015; Rahman et al., 2019). In 2012, CHIKV was reported for the first time in Bhutan (Wangchuk et al., 2013). Further, Nepal reported the upsurge of CHIKV during the period 2013–2015 (Pandey et al., 2017). In Pakistan, although the first detected case was in December 2016, anti-CHIKV antibodies in human sera and rodents have been detected as early as 1980s (Darwish et al., 1983). This virus was introduced into Zhejiang and Tianjin province of China by travelers from Bangladesh to Myanmar in 2017, respectively (Pan et al., 2019; Xie et al., 2020). Again, a study conducted in India through July 2018, involving testing a total of 1549 samples, almost 50% patients tested positive for CHIKV infection (Badar et al., 2019). Although, CHIKV infection dates back to 1950s and has started spreading its roots quite consistently since 2005 yet absence of a nationwide surveillance system for such arboviral diseases and its reported misdiagnosis as Dengue could be one of the reasons for the lack of a detailed prevalence studies. Further, evidence for chikungunya-dengue co-infection has been found in Angola, Gabon, India, Madagascar, Malaysia, Myanmar, Nigeria, Saint Martin, Singapore, Sri Lanka, Tanzania, Thailand, and Yemen which adds to the confusion (Furuya-Kanamori et al., 2016).

### Epidemiology of CHIKV in India

**FIGURE 3:**
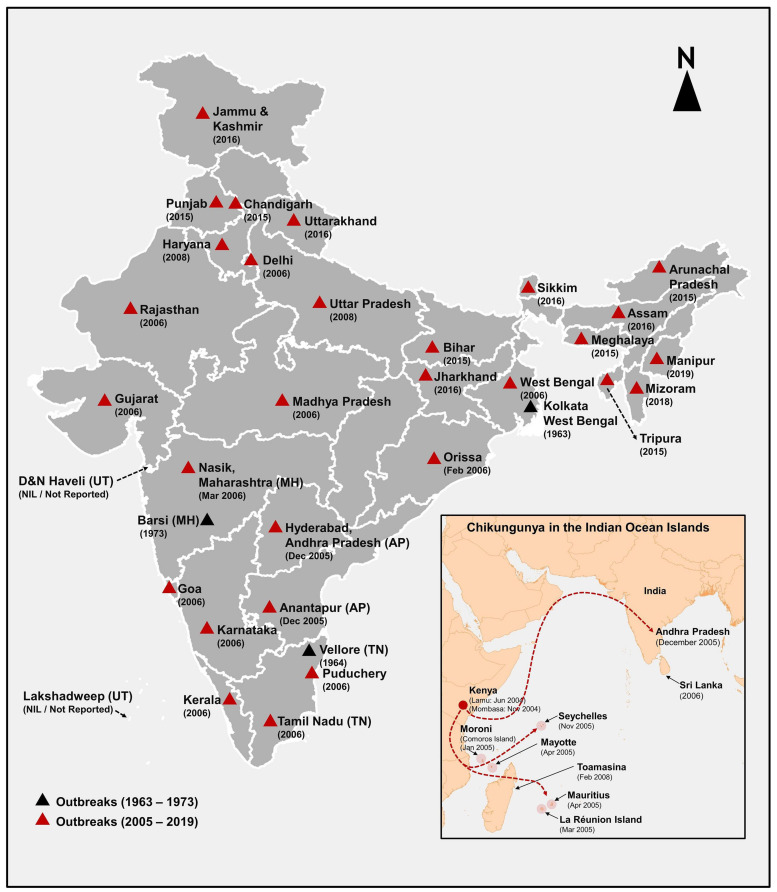
Reported chikungunya outbreaks in India and Indian Ocean Islands (IOI) shows the year of onset of chikungunya outbreaks between 1963 and 2019 in India and the change in genotype from Asian (during the 1963–1973) to ECSA (2005–2019) is sketched in black and red triangles. The data source for generating. This figure is from NVBDCP (2014-15); National Health Profile (2019); National Center for Disease Control (2020).

Chikungunya outbreaks have been reported from India during the period 1963–1973 and 2005–2019. The first CHIKF outbreak in India was reported from Kolkata (Calcutta), West Bengal, in 1963 (Sarkar et al., 1964; Shah et al., 1964; Sudeep and Parashar, 2008; Figure 3). However, retrospective serological studies have shown that chikungunya existed in India prior to 1963 (Pavri, 1964; Banerjee, 1965). In 1964, Chennai (Thiruvengadam et al., 1965), Pondicherry, and Vellore reported outbreaks of chikungunya (Jadhav et al., 1965; Naresh Kumar and Sai Gopal, 2010). In 1965, chikungunya outbreaks were reported from Visakhapatnam, Kakinada, Rajahmundry and Nagpur, in 1973 in Barsi in Maharashtra (Rodrigues et al., 1972; Padbidri and Gnaneswar, 1979; Yergolkar et al., 2006) and some authors have opined that lack of surveillance between 1973 and 2005 might have been the reason for lack of CHIKV reports (Pavri, 1986; Ravi, 2006). However, the re-emergence of the virus could be due to several other reasons (Mishra and Ratho, 2006). CHIKV is known to have three genotypes, namely, West African (WA), Asian and East Central South Africa (ECSA) genotypes and the circulating virus in India until the 1970s have been reported to be of the Asian genotype (Kalantri et al., 2006; Naresh Kumar and Sai Gopal, 2010).

### CHIKV in India Post-2005

After the emergence of CHIKV in the Indian Ocean islands in 2005, CHIKV re-emerged in India after 32 years (Lahariya and Pradhan, 2006), affecting Hyderabad and Ananthapur district of Andhra Pradesh in South India and eventually affected 1.4 million people in 13 states (Schuffenecker et al., 2006) with huge economic and productivity loss of 391 million rupees (Krishnamoorthy et al., 2009). Ahmedabad city of Gujarat and Kerala state were the worst affected places (Sudeep and Parashar, 2008). The 2005–2006 outbreak in India was caused by the ECSA genotype (Kalantri et al., 2006; Naresh Kumar and Sai Gopal, 2010). The E1-A226V mutation of the virus and their adaptation to the *A. albopictus* resulted in increased susceptibility among pediatric population, neurological complications, as well as mortality associated with this outbreak (Naresh Kumar and Sai Gopal, 2010).

**FIGURE 4:**
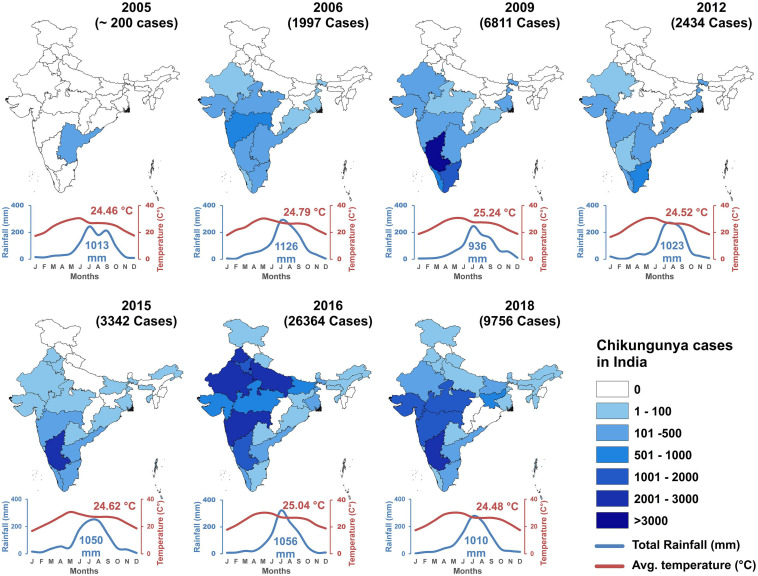
Year-wise CHIKF cases in India with annual rainfall and temperature (2005–2018).

In the 2005–2006 outbreak, 13 states of India reported CHIKF. In 2009, the infection expanded to 15 states in India. The number of states which reported CHIKF increased to 23 by the year 2015, 28 states in 2016, and 30 states/Union territories in the year 2019. Jammu and Kashmir, Mizoram, and Manipur has reported ≤10 laboratory confirmed CHIKF cases until December 2019. Union territories of Dadra and Nagar Haveli and Lakshadweep has not reported CHIKF cases until now. The year-wise prevalence of CHIKF cases in India from 2005 to 2018 along with temperature and rainfall data is shown in Figure 4. Maximum number of laboratory- confirmed cases were reported in the year 2016, followed by 2017, and 2019. Highest confirmed cases were reported in Karnataka, Delhi, and Maharashtra (Supplementary Table 1). Available data on year-wise percentage positivity of CHIKV between 2014 and 2019 show some variation across the different seasons in India. However, there seem to be a general trend of lower percentage positivity values recorded during the summer periods which increases as rainy season sets in and continues to be high through winter (Department of Health and Family Welfare, Government of India, 2018; National Center for Disease Control, 2020) (Supplementary Figure 2). In 2019, a total of 81914 cases were clinically suspected for CHIKV, out of which, 12205 (14.9%) laboratory confirmed chikungunya was reported in 21 Indian states and 3 Union territories in this year. Majority of the CHIKV cases were reported from Karnataka (3664), followed by Maharashtra (1646), Telangana (1358), and least was reported from Uttarakhand (1) (Supplementary Figure 3). In 2020, a total of 18533 cases were clinically suspected for CHIKV and 2812 (15.2%) cases were laboratory confirmed as CHIKF until July 2020 (Department of Health and Family Welfare, Government of India, 2018). Currently, CHIKF is endemic in 24 Indian states and 6 union territories indicating that this as an important health problem in our country (NVBDCP, 2014-15).

### Disease Transmission and Vector Biology

Chikungunya virus is transmitted mainly through Aedes mosquitoes. Mosquitoes acquire CHIKV when it bites the infected human with viremia. During this extrinsic incubation period in the mosquito, CHIKV replicates in the midgut, circulates through body cavity, reaches to the salivary gland, establishes infection, and secretes into the saliva. CHIKV reaches to new host when mosquito feeds on their blood. The transmission cycle continues, virus gets amplified in the new host again picked up by mosquito if they feed on the infected person and the transmission cycle goes on.

The most common transmission mechanism of CHIKV is the horizontal transmission through the mosquito saliva. Mosquito injects the saliva carrying virus to a new host when it feeds on their blood. However, a low rate of vertical transmission may also occur through infected eggs (Agarwal et al., 2014). Entomological survey conducted by Jain et al. (2016) has demonstrated the presence of vertical transmission in *Aedes aegypti* population. Agarwal et al. (2014) confirmed experimental vertical transmission of ECSA genotype of CHIKV in *A. aegypti* from India. Studies have documented vertical transmission of Indian Ocean Lineage of CHIKV up to F5 and F6 progenies in *A. aegypti* and *A. albopictus* (Chompoosri et al., 2016). Likewise, vertical transmission in the host has also been documented in the global scenario. Mother-to-child transmission has been reported in women who developed CHIKF in the last week of gestation prior to delivery (Gerardin et al., 2008; Fritel et al., 2010). One case study from Brazil has documented probable vertical transmission leading to maternal and neonatal death. Hence, clinicians must consider the possibility of neurological complications and find ways to treat this (Bandeira et al., 2016; Cardona-Correa et al., 2017; Oliveira et al., 2018). The risk of transmission appears to be rare before 22 weeks of gestation, however, the pathogenic mechanism is not clear yet (Lenglet et al., 2006). Overall, vertical transmission is linked with poor pregnancy outcomes in some cases (Robillard et al., 2006; Senanayake et al., 2009). In the contrary, vertical transmission of CHIKV has not been documented till date in the Indian scenario (Nigam et al., 2016).

### Molecular Epidemiology

Chikungunya virus genome consists of a 11.8 kb linear, single-stranded, positive-sense RNA molecule., organized into two open reading frames (ORFs) encoding non-structural (nsP1-nsP2-nsP3-nsP4) and structural (C-E3-E2-6K-E1) polyproteins at the 5′ and 3′ ends, respectively. These ORFs are flanked by 5′ and 3′ untranslated regions which regulate viral gene expression, replication, translation and host-virus interactions thereby significantly impacting viral evolution, pathogenesis and host range (Hyde et al., 2015). Mutations at these proteins have shown to attribute viral fitness and vector preferences (Tsetsarkin et al., 2007, 2011a,b, 2014; Casal et al., 2015; Agarwal et al., 2016; Jain et al., 2016). Continuous surveillance of phylogeny and mutations of the circulating viral strain is very important for early detection of mutation in the viral genome and evolution of new strain and that can help us to prepare for the prevention and control of future epidemics.

Phylogenetic analysis of CHIKV based on whole genome and individual genes grouped the virus into three genotypes: East/Central/South African, West African, and the Asian (Powers et al., 2000). A recent study favored further the division of ECSA into three subgroups: ECSA I, ECSA II, and ECSA III, based on the Bayesian analysis, and E1 gene sequences (Casal et al., 2015).

Starting from 2005, there were reports of the ECSA genotype causing severe epidemics from Kenya to India where over one million cases were recorded and is said to be massively under estimated (Mavalankar et al., 2007). Subsequent studies have also identified the ECSA genotype as the cause of outbreaks in India (Niyas et al., 2010; Agarwal et al., 2019; Tandel et al., 2019; Jain et al., 2020). A recent study revealed the co-circulation of two distinct subclades of ECSA in South India when the circulating strains were characterize from the 2016 outbreak across the country (Harsha et al., 2020). One of these subclades had been earlier reported in 2010 from an outbreak in Delhi (Shrinet et al., 2012). Studies on CHIKV microevolution during the 2005–2006 Indian Ocean epidemic revealed the presence of E1-A226V mutation (Schuffenecker et al., 2006) which was later shown to mediate improved fitness of CHIKV in *A. albopictus* regarding midgut infectivity, transference to salivary glands and subsequent transmission to the vertebrate host (Tsetsarkin et al., 2007). However, a number of Indian studies have reported the absence of this mutation (Agarwal et al., 2019). Essentially, apart from the Kerala study (Niyas et al., 2010) which reported the E1-A226V strain, many other studies have found this mutation to be absent in their isolates. Consequently, other mutations that aid increased fitness in *A. albopictus* may be responsible for the dominance of ECSA in Indian outbreaks. Since 2006, *A. albopictus* has been recognized as the main vector in some parts of India, the Indian Ocean, Thailand, Malaysia, Singapore, Gabon, Sri-Lanka and Italy (Tsetsarkin et al., 2011a). However, a number of mutations in the CHIKV genome were discovered to have epistatic effect (lineage specific) on the E-A226V mutation. For instance, E2-211I in most ECSA strains and E1-98T in all endemic Asian strains tend to block adaptation to *A. Albopictus* (Tsetsarkin et al., 2009). Whether these mutations that result in epistasis are playing a significant role in controlling the frequency of outbreaks has not been deliberated. It was speculated previously that it would be interesting to evaluate the effect of these mutations with respect to the presence or absence of the E1-A226V mutation (Shrinet et al., 2012). However, it was suspected that the characteristic inter-epidemic quiescence of CHIKV outbreaks might be partly attributed to the effect of epistatic mutations on important adaptive changes in the CHIKV genome (Weaver et al., 2021). In 2010, a novel non-conservative mutation, E2-L210Q was discovered in both human and mosquito samples in Kerala (Niyas et al., 2010). Laboratory investigations revealed that E2-L210Q acts primarily at the level of infection of *A. albopictus* midgut epithelial cells, thus providing additional selective advantage that may facilitate even more efficient virus circulation and persistence in endemic areas (Tsetsarkin et al., 2011b). Considering the fact that *A. albopictus* was largely responsible for outbreak in Kerala (Niyas et al., 2010), it is plausible to suggest that the E2-L210Q mutation further enhanced the vector competence, thus driving the epidemic.

Two novel mutations, E1-K211E and E2-V264A were also identified together with molecular signatures that suggest possibility of recent recombination events in Indian CHIKV isolates in an outbreak in Delhi (Shrinet et al., 2012). A study had earlier asserted that homologous recombination did not play a role in the genetic diversity of Asian strains albeit two potential recombination events were found (Cui et al., 2011). However, Casal et al. (2015) proved that recombination was an additional mechanism of CHIKV genetic diversity when it was demonstrated that the recombinant strain KJ679578/2011 (ECSA III) was derived from ECSA III and ECSA I. A more recent study, while highlighting the role of 3′ UTR plasticity in CHIKV evolution, has also proposed that recombination in CHIKV accelerates adaptability (Filomatori et al., 2019; Weaver et al., 2021). Interestingly, these mutations in E1 and E2 (E1-K211E and E2-V264A) have been shown to enhance the fitness of *A. aegypti* resulting in 13-fold increase in infectivity; 15-fold enhancement in dissemination; and a 62-fold surge in transmission (Agarwal et al., 2016). Notably, *A. aegypti* is predominant in Delhi, hence the active role of this mutation causing the outbreak within the metropolis is apparent. In the previous study that compared the 2010 and 2016 outbreaks in India, a number of mutations were identified, including the E2-G55R, H73Y protein which was unique to the 2010 outbreak and associated with higher disease severity; and the E1-I317V which was present in all the sequenced 2016 samples (Jain et al., 2020). Some studies have also identified several mutations in CHIKV during epidemics and sporadic infections in India (Table 2). Some of these mutations have been recognized as having high evolutionary potential, higher disease severity (Jain et al., 2020), and capacity to cause epidemics (Singh et al., 2012). Interestingly, some Indian studies have recorded a 100% presence of these mutations including E1-K211E, M269V, and D284E in their sequenced isolates (Mudurangaplar and Peerapur, 2016; Tandel et al., 2019).

**TABLE 2:** Few CHIKV mutations reported between 2010 and 2020.

Location	Period	Protein	Mutation	References
Delhi	2010	E1	**K211E M269V D284E** V179A S234P R196K R247C	Agarwal et al. (2016)
		E2	V50A C389R **A164T I211T** **V264A S299N T312M A344T** **S375T V386A**	
			G55R H73Y	Jain et al. (2020)
		E3	**V42I P59S**	
		nsP1	G230R M314L R85H T215A F391L K128T V172L V452A	
		nsP2	G641D M290T A256V V639I	
		nsP3	I175V STOP524R	
		nsP4	G85R	
	2016	C	Q58R	
		E1	**I317V** N348I S167P T288I	
		E2	K189R L412F	
		nsP1	K224T	
		nsP2	**H130Y** E145D T446S P689S H109Q	
		nsP3	H377R	
		nsP4	S55N	
		E1	R123K T145S V179M K211E P232Q I261D M269V D284E I317V/M V322A N389K	Kaur et al. (2017)
	2016	E1	K211E M269V D284E T145A N149K G150D G314A	Tandel et al. (2019)
Central India	2016–2017	E1	K211E M269V D284E I317V V322A	Agarwal et al. (2019)
Southern India	2011–2014	E1	**K211E M269V D284E**	Mudurangaplar and Peerapur (2016)
Tamil Nadu and Andhra Pradesh	2009–2010	E1	K211E P58L G195R	Sumathy and Ella (2012)
		E2	**V264A** K47N G55R K66E	
		E3	D40N	
		C	A232V	

*Highlighted: 100% of sequenced isolated show this mutation.*

Jain et al. (2020) also reported the presence of mutations in nsP1 (G230R) and nsP3 (opal 524R) proteins which were unique to the 2010 outbreak and were associated with higher disease severity. Apart from that, one nsP2 mutation, H130Y, was present in all the sequenced 2016 samples (Jain et al., 2020). It is very important to note that a combination of mutations in the nsP1 protein (G230R and K299E) resulted in CHIKV resistance to 6′-β-Fluoro-Homoaristeromycin and 6′-Fluoro-Homoneplanocin A, which are carbocyclic adenosine analogs and had initially displayed potent antiviral activity on the wild-type virus (Kovacikova et al., 2020). These mutations might have contributed to the difficulties that is currently faced in research toward CHIKV vaccines and therapeutics. A number of other combinations of mutations, some of which have been mentioned in this review, have also been studied and found to increase the fitness of the virus. These include a combination of mutations in the nsP1 and the opal stop codon preceding in the nsP3 protein. Mutations in these regions including nsP3 (opal 524R) - nsP1 (G230R); nsP3 (opal 524R) - nsP1 (V326M); and nsP3 (opal 524R) - nsP1 (G230R) - nsP1 (V326M), have been shown to confer resistance to polyamine depletion in addition to enhanced viral replication (Mounce et al., 2017). Although the authors concede that individual mutations do not induce polyamine depletion resistance, it is still worrisome because some of these combinations have already been reported in Indian outbreaks (Jain et al., 2020).

These reported mutations and further experiments to determine their role in vector range and competence; fitness in vector; disease transmission and severity, have shown that CHIKV evolution has been a significant driving force of epidemics in India. This is evidenced by the current surge in the number of epidemics witnessed in recent years since 2010 despite the under-reporting for obvious reasons. The ability to adapt to a new vector and expand its geographical distribution by a single point mutation as observed in E1-A226V makes CHIKV a dynamic pathogen that has potential to attain a global public health concern.

## Antiviral and Vaccine Initiatives

Currently, there is no licensed therapeutics or vaccines for the treatment of CHIKV infections in the market. However, this section describes various efforts at different stages of development, both of therapeutics and vaccines against CHIKV.

### Vaccines

Vaccines have a background marked by the beginning in the late 18th century across the globe (Plotkin, 2014) and India was amongst few countries to be involved in these efforts. First CHIKV episode during 1960s prompted the start of research for the development of CHIKV immunization (Halstead et al., 1963). Since then, the quest for CHIKV vaccine candidates that balances the immunogenicity and provides adequate safety to CHIKV pathogenicity continues (Gao et al., 2019).

The very first live attenuated vaccine (LAV) of CHIKV was made in 1986 from Thailand isolate AF15561. LAV was known as 181/clone25 and it was made through 18 plaque passages in Human lung cells (MRC-5). This antibody was profoundly immunogenic in humans, however, it led to arthralgia in few cases (Edelman et al., 2000). Roques et al. (2017) developed vaccine candidates like MVA-CE and DREP-E in non-human primate model that targeted the nsP3 envelope and capsid using ECSA strain of Indian Ocean lineage. They were able to generate neutralizing antibodies against an isolate of the Asian Genotype. The role of nuclear localization sequence (NoLS) in the N-terminal region of capsid protein was characterized for the generation of LAVs. CHIKV infection was effectively neutralized when serum from CHIKV-NoLS-immunized mice was introduced, and the infectious titer of CHIKV-NoLS was found to be insensitive to freeze-thaw cycles, unlike CHIKV-WT, thus demonstrating preclinical safety and stability of CHIKV-NoLS (Taylor et al., 2017; Abeyratne et al., 2018). A few LAVs have been designed based on multiple replacements of synonymous codons thereby leading to decrease in the virus mutational robustness. The approach considerably reduces the risk of reversion because it involves hundreds of synonymous modifications to the genome, making such viruses as promising vaccine candidates (Carrau et al., 2019). Production of LAVs by mutation of non-structural proteins was also investigated by Chan et al. (2019). They found R532H mutation in nsP1 caused reduced infectivity in mouse tail fibroblasts but type-I IFN response was enhanced compared to WT-CHIKV (Chan et al., 2019). Recently, in a novel attempt to generate LAV candidate for CHIKV with complete deficiency of capsid, a single dose of the virus was reported to provide complete protection upon challenge with wild-type CHIKV in mice (Zhang et al., 2019).

Inactivated vaccines are considered to be secured and safe. Initially, researchers attempted to generate CHIKV vaccines by inactivating the virus by the means of heat or chemical treatment. The very first CHIKV vaccine was a formalin inactivated virus prepared from an assortment of cell like chick embryo cells and African green monkey kidney cells way back in 1972 (Eckels et al., 1970; White et al., 1972). Although, the formalin inactivated vaccine induced neutralizing antibody responses successfully and was effective in protecting mice against an intracerebral challenge, but it did not induce potent protective immune response in human volunteers (Harrison et al., 1971). Tiwari et al. (2009) had a trial where inactivated vaccine was produced in Vero cells and the titer of neutralizing antibodies peaked at 6–8 weeks post vaccination.

One of the most successful strategies in the field of vaccine development is the use of virus vectors that involves use of less pathogenic viruses to deliver genetic materials into living cells. In case of CHIKV vaccine development, the CHIKV structural genes are inserted into the genome of the virus vector that initiates the expression of CHIKV structural proteins or contains CHIKV structural proteins embedded in the virion (Ramsauer and Tangy, 2016). A new measles virus vectored vaccine (MV-CHIK) was tested for its efficacy in preventing CHIKV infection in a non-human primate model and found to be protective when challenged with the virulent La Reunion CHIKV strain. These results further documented the immunogenicity and efficacy of such vaccines that exhibited promising results in Phase I-II clinical trials (Reisinger et al., 2019; Rossi et al., 2019).

Another popular technique utilized in CHIKV vaccine development is the use of chimeric vaccine. To defeat the reversion capability of generally attenuated alphavirus vaccine and to address the developing demand to prevent CHIKV infections, chimeric approach has been employed for the development of vaccine. Using the structural genes of CHIKV and either a naturally attenuated strain of eastern equine encephalitis virus (EEEV), Venezuelan equine encephalitis attenuated vaccine strain TC-83, or Sindbis virus, chimeric alphavirus vaccine candidates were developed. The vaccinated mice were found to be fully protected against the disease, characterized by high titers of neutralizing antibodies (Wang et al., 2008; Plante et al., 2011). In another approach, chimeric virus was designed with insect specific alphavirus Eilat virus (EILV), and CHIKV structural proteins. Although, it mimicked the early stages of CHIKV replication in vertebrates, yet, it remained completely defective for viral productive replication, thus, providing a high degree of safety and robust immunity against viremia (Erasmus et al., 2017).

Couderc et al. (2009) purified human polyvalent immunoglobins from plasma samples of patients in the convalescent phase of CHIKV infection and investigated its curative effect in CHIKV mouse models. These immunoglobulins were found to contain anti-CHIKV antibodies and exhibited *in vitro* neutralizing activity and *in vivo* prophylactic and therapeutic efficacy. Hence, this approach can provide a safe preventive strategy for CHIKV infected individuals (Couderc et al., 2009). The immunogenic potential of recombinant proteins has been reported with time. Khan et al. (2012) expressed the CHIKV envelope protein in *E. coli* and found that the recombinant protein not only retained their antigenicity and immunogenicity but also induced production of neutralizing antibodies and cell mediated immune response in immunized mice.

Virus like particles (VLPs) as a vaccine candidate has been evaluated *in vitro* and *in vivo* in Balb/C mice and induction of both humoral and cellular immunity were observed. Neonates immunized with anti-CHIKV-VLP antibodies were protected from CHIKV infection, establishing VLP as a promising vaccine candidate (Saraswat et al., 2016).

Many advancements in molecular and biochemical technologies have led to the adoption of various strategies to develop vaccines as summarized in Table 3.

**TABLE 3:** List of possible CHIKV vaccine candidates.

Vaccine	Target	Type of vaccine	Status	References
**Global initiatives**				
T-E CHIKV	Lipid containing envelope of the virus	Inactivated Vaccine; 1st generation	Preclinical	Eckels et al. (1970)
USPHSRPB	Structural and envelope proteins	Inactivated Vaccine; 1st generation	Phase I	Harrison et al. (1971)
USPHSRPB	Structural and envelope proteins	Inactivated Vaccine; 1st generation		White et al. (1972)
CHIK181/clone 25	Reversion of attenuating point mutations	LAV; 1st generation	Preclinical	Levitt et al. (1986)
TSI-GSD-218	Inhibition of viral replication	LAV; 1st generation	Phase II	Edelman et al. (2000)
181/c25	Reversion of attenuating point mutations	Chimeric virus; 3rd generation	Preclinical	Wang et al. (2008)
CHIKVIg-01	Prevention of viral dissemination in tissues	Subunit Vaccine; 2nd generation	Preclinical	Couderc et al. (2009)
CMB/R	Envelope proteins	VLP; 2nd generation	Preclinical	Akahata et al. (2010)
CHIKV/IRES	Targets viral entry	Recombinant Vaccine; 3rd generation	Preclinical	Plante et al. (2011)
VEE/IRES-C/CHIKV	nsP2 envelope and capsid	Chimeric virus; 3rd generation	Preclinical	Wang et al. (2011)
pMCE321	E1 and E2 glycoproteins	DNA vaccine; 3rd generation	Preclinical	Mallilankaraman et al. (2011)
dMAb	CHIKV envelope	DNA vaccine; 3rd generation	Preclinical	Muthumani et al. (2016)
EILV/CHIK	EILV cDNA clone containing CHIKV structural proteins	Chimeric virus; 3rd generation	Preclinical	Erasmus et al. (2017)
MVA-CE	nsP3 envelope and capsid	LAV;1st generation	Preclinical	Roques et al. (2017)
DREP-E	nsP3 envelope and capsid	LAV;1st generation	Phase II	Roques et al. (2017)
CHIKV-NoLS	N-terminal region of capsid protein	LAV;1st generation	Preclinical	Taylor et al. (2017), Abeyratne et al. (2018)
MV-CHIK	Measles vectored CHIKV structural proteins	Vector virus; 3rd generation	Preclinical	Reisinger et al. (2019), Rossi et al. (2019)
CHIKV	Multiple synonymous mutations in genome to reduce mutational robustness	LAV; 1st generation	Preclinical	Carrau et al. (2019)
SuperStop	Multiple synonymous mutations in genome to reduce mutational robustness	LAV; 1st generation	Preclinical	Carrau et al., 2019)
Stop CHIKV	Multiple synonymous mutations in genome to reduce mutational robustness	LAV; 1st generation	Phase I	Carrau et al. (2019)
15nsP3	E515V-nsp2	LAV; 1st generation	Preclinical	Chan et al. (2019)
1C-CHIKV	Capsid deletion	LAV; 1st generation	Preclinical	Zhang et al. (2019)
RHEV-CHIKV	E515V-nsp2	LAV; 1st generation	*In vitro*	Chan et al. (2019)
**Indian initiatives**				
CK1/2	Viral replication	Vector virus; 3rd generation	*In vitro*	Dash et al. (2008)
CHIK-FI	Envelope polyprotein	LAV; 1st generation	Preclinical	Tiwari et al. (2009)
rCHIKE1/E2	E1 envelop protein	Subunit; 2nd generation	*In vitro*	Khan et al. (2012)
CHIKV-VLPs	Structural proteins introduced into yeast expression system	VLP; 2nd generation	Preclinical	Saraswat et al. (2016)
VSV1G-CHIKV	E3-E2-6K-E1 envelope polyprotein	Vector virus; 3rd generation	Preclinical	Subudhi et al. (2018)

As a result of the urgent need for vaccines against various viral infections, Government of India started National Immunization Program under which various licensed vaccine manufacturing units were set up in the country. In the beginning of twentieth century, vaccine institutes were setup in India, which eventually moved toward privatization. Bharat Biotech, Indian.

Immunological, Defense Research Development Establishment, National Institute of Virology, Serum Institute of India are few Indian companies/organizations working on CHIKV vaccine dedicatedly.

In the quest to develop an acceptable vaccine against CHIKV, certain considerations must be followed. First, the vaccine titer should be enhanced so that it provides better protection. Second, the future vaccines should provide long term protection. However, short term protection is also required to control local outbreaks. Third, the dose of vaccine would be such that there should not be significant difference in the response to a vaccine owing to the complexity of population (different age groups). Fourth, the vaccine candidates that are effective in animal models should be taken forward for human trials. Fifth, the cost of production of vaccine should be cheap and affordable. Sixth, the vaccines should be thermostable, unlike RNA, so that it could be easy for transport and storage. Lastly, their administration should be convenient so that it does not cause any discomfort or side effects post vaccination. Hence, it can be assumed that with the employment of new strategies and new studies, commercially cost effective CHIKV vaccine with high safety and strong immunogenicity can be developed in the near future.

### Antivirals

Albeit different antivirals are accessible for different viral diseases, there are no approved antivirals against CHIKV diseases. Hence, the current therapies mostly involve in management of symptoms using non-salicylate analgesics and non-steroid anti-inflammatory drugs (NSAIDS) (Thiberville et al., 2013). A number of known antiviral agents and other novel compounds have been examined for their activity against CHIKV and many show promises.

Ribavirin and 6-Azauridine show antiviral activity in a concentration dependent manner and found to reduce cytopathic effect and virus titer on infected Vero cells (Briolant et al., 2004). Arbidol a broad-spectrum antiviral drug showed potent inhibitory activity against CHIKV when examined on Vero cells and primary human fibroblasts (IC_50_ < 10 μg/ml) (Delogu et al., 2011). Furin inhibitors have been reported as potent inhibitors of CHIKV almost comparable to chloroquine (Ozden et al., 2008). CHIKV is highly sensitive to antiviral activity of Type I interferons (IFN-α/β), mediated by 2’,5’-oligoadenylate synthetase (OAS-3) family of proteins (Brehin et al., 2009).

Diterpenoids like prostratin and 12-O-tetradecanoylphorbol 13-acetate (TPA) exhibited antiviral activities in virus-cell-based assays against CHIKV as well as other alphaviruses (Bourjot et al., 2012). Polyinosinic acid (Poly I:C), mimic of double-stranded RNA is recognized by toll like receptor-3 (TLR-3) leading to induction of interferons in many cell types. TLR-3 pathway is suggestive of inducing innate immune responses against many viruses including CHIKV (Li et al., 2012). Since CHIKV infection is usually cleared within 7–8 days post infection before adaptive immune response emerges, therefore innate immune response becomes important for CHIKV clearance.

Several studies have also examined different compounds including viperin; resazurin; human monoclonal antibody C9:also many human and mouse anti-CHIKV monoclonal antibodies have been found protective *in vivo*; favipiravir; Retinoic acid inducible gene I (RIG-I); Various NSAIDS like mefenamic acid in combination with common antiviral drug like ribavirin; sulfonyl amidines and flavaglines (FL3 and FL23); imipramine; Curcumin; a series of arylalkylidene derivatives of 1, 3-thiazolidin-4-one; chloroquine; and ribavirin (Dash et al., 2008; Ravichandran and Manian, 2008; Padmakumar et al., 2009; Khan et al., 2012; Teng et al., 2012; Cruz et al., 2013; Parashar et al., 2013; Rathore et al., 2013; Selvarajah et al., 2013; Chopra et al., 2014; Delang et al., 2014; Olagnier et al., 2014; Albulescu et al., 2015; Jadav et al., 2015; Wintachai et al., 2015; Rothan et al., 2016; Mounce et al., 2017; Wichit et al., 2017; Subudhi et al., 2018; Jin and Simmons, 2019; Martins et al., 2020; Dong and Dimopoulos, 2021) and reported promising activity against CHIKV as summarized in Table 4. Lack of licensed antivirals or vaccines for CHIKF makes it yearning for the development of potent drugs against it.

**TABLE 4:** Showing tested antiviral drugs against CHIKV.

Antivirals	Target	Assay type	References
**Global initiatives**			
6-Azauridine	Inhibition of orotidine monophosphate decarboxylse enzyme	*In vitro* (vero cells)	Briolant et al. (2004)
Chloroquine	Inhibition of fusion of the viral E1 protein with endosomal membrane by raising the endosomal pH	*In vitro* (vero cells) and *in vivo (macaque models)* Clinical trials in CHIKV infected Humans	Roques et al. (2018)
Furin inhibitors	Inhibition of virus maturation via inhibition of cellular furins	*In vitro* (Human muscle satellite cells)	Ozden et al. (2008)
Oligoadenylate synthetase (OAS-3)	Inhibits CHIKV replication through RNase L dependant pathway	*In vitro* (HeLa cells)	Brehin et al. (2009)
Arbidol	Inhibits CHIKV replication through single amino acid substitution	*In vitro* (vero cells)	Delogu et al. (2011)
5,7-Dihydroxyflavones	Unidentified target and mechanism of action	*In vitro* (BHK replicon cell line)	Pohjala et al. (2011)
Prostratin and TPA	Activation of protein kinase C	*In vitro* (vero cells)	Bourjot et al. (2012)
Polyinosinic acid	Stimulation of IFN-α and β and antiviral genes	*In vitro* (BEAS-2B cells)	Li et al. (2012)
Viperin	Targets endoplasmic reticulum	*In vivo* (monocytes)	Teng et al. (2012)
Resazurin	Inhibition of kinases involved in apoptosis	*In vitro* (HuH-7 cells)	Cruz et al. (2013)
Monoclonal antibody C9	Interaction with CHIKV E2 glycoproteins	*In vitro* (vero cells) *In vivo* (C57/BL6 mice)	Selvarajah et al. (2013)
Favipiravir	Inhibition of viral genome replication	*In vitro* (vero cells) *In vivo* (AG129 mice)	Delang et al. (2014)
RIG-1 agonists	Stimulation of immune response	*In vitro* (MRC-5 cells)	Olagnier et al. (2014)
Suramin	Inhibition of CHIKV RNA synthesis	*In vitro* (BHK-21, and Vero-E6 cells)	Albulescu et al. (2015)
Ribavirin	Inhibition of viral genome replication via GTP pool depletion	*In vitro* (vero cells) *In vivo* (ICR mice)	Rothan et al. (2015)
Flavaglines	Interference with the binding of CHIKV Prohibitin-1	*In vitro* (HEK-293T cells)	Wintachai et al. (2015)
Benzouracil-Coumarin-Arene conjugates	Unidentified target and mechanism of action	*In vitro* (vero A cells)	Hwu et al. (2015)
Mefenamic acid in combination with ribavirin	Inhibition of viral replication	*In vitro* (vero cells) *In vivo* (ICR mice)	Rothan et al. (2016)
Imipramine	Inhibition of viral replication	*In vitro* (vero and HFF1 cells)	Wichit et al. (2017)
Curcumin	Inhibition of viral binding at the cell surface	*In vitro* (HeLa, BHK-21, and Vero-E6 cells)	Mounce et al. (2017)
Chloroquine	Inhibition of viral replication	*In vitro* (primary macrophages and fibroblasts cells) *in vivo* (cynomolgus macaques)	Roques et al. (2018)
**Indian initiatives**			
Ribavirin	Inhibition of viral genome replication	Clinical trials in CHIKV infected Humans	Ravichandran and Manian (2008)
Aceclofenac in combination with hydroxychloroquine and prednisolone	inhibition of synthesis of prostaglandins and having chondroprotective effect	Humans	Padmakumar et al. (2009)
Chloroquine	Inhibition of fusion of the viral E1 protein with endosomal membrane by raising the endosomal pH	*In vitro* (vero cells)	Khan et al. (2010), Chopra et al. (2014)
Mycophenolic acid (MPA)	Depletion of intracellular guanosine pool	*In vitro* (vero cells)	Khan et al. (2011)
Si-RNAs	Inhibition of protein synthesis by targeting nsp1 and E2	*In vivo* (swiss albino and C57/BL6 mice)	Parashar et al. (2013)
HSP-90 inhibitors (HS-10 and SNX-2112)	Interaction with CHIKV nsp3 and nsp4	*In vitro* (HEK-293T cells) *In vivo* (SvA129 mice)	Rathore et al. (2014)
Thiazolidine derivative	Inhibition of CHIKV nsp2 protease activity	*In vitro* (vero cells)	Jadav et al. (2015)

### Novel Drug Repurposing Initiatives Against Chikungunya

Lots of existing drugs are being repurposed for fighting against CHIKV as they have well established formulations, well known clinical trial safety data. Novel drug discovery requires enormous amount of time, money, and effort. Success rate of drug repurposing or drug repositioning is relatively high because the target drugs have already been tested for their efficiency against other diseases and have been proved to be safe for human use. Also, the existing drugs against CHIKV have achieved only limited success with questionable therapeutic effectiveness (Subudhi et al., 2018). Considering the extensive research in this field, it is worthwhile to mention a list of few drugs available for repurposing and the rest are listed in the table below (Table 5).

**TABLE 5:** List of suggested novel antivirals and repurposing initiatives against CHIKV.

Drugs	Targeted virus	Assay type	References
Seco-pregnane steroids	TMV, SINV	*In vivo*	Li et al. (2007)
Naringenin, Apigenin	CHIKV	*In vitro*	Pohjala et al. (2011)
Lupenone, β-amyrone	CHIKV, DENV	*In vitro*	Bourjot et al. (2012)
Baicalin, Quercetagetin	CHIKV	*In silico*	Seyedi et al. (2016)
Hesperetin	CHIKV	*In silico*	Oo et al. (2016)
Doxycycline	CHIKV	*In silico*	Rothan et al. (2016)
Pimozide, Cerulenin, and Tivozanib	CHIKV	*In vitro* *In vivo*	Karlas et al. (2016)
Auranofin	CHIKV	*In vitro* *In vivo*	Langsjoen et al. (2017)
Acrylamides derivative (LQM334)	CHIKV	*In silico*	Passos et al. (2020)
**Indian Initiatives**			
Harringtonine	CHIKV	*In vitro*	Kaur et al. (2013)
Aplysiatoxin	CHIKV	*In vitro*	Gupta et al. (2014)
CID-5808891	CHIKV	*In silico*	Agarwal et al. (2015)
Picolinate	CHIKV	*In silico*	Sharma et al. (2016)
Ribostamycin sulfate	CHIKV	*In silico*	Kumar et al. (2019)
Dihydrorugosa flavonoids	CHIKV	*In silico*	Puranik et al. (2019)
Rutin, Moralbanone, and Kaempferol	CHIKV	*In silico*	Khan et al. (2020)

#### Seco-Pregnane Steroids

Seco-pregnane steroid glaucogenin C and its monosugar glycoside cynatratoside A have been shown to be non-toxic to host cells while selectively inhibiting alphavirus-like positive-strand RNA virus (Li et al., 2007).

#### Baicalin

Compared to other ligands, baicalin has been identified as a potential inhibitor of viral activity by showing a good binding affinity (−9.8 kcal/mol) (Seyedi et al., 2016).

#### Hesperetin

Hesperetin, a bioflavonoid, exhibits inhibitory effect on multiple viruses *in vitro*. Results from *in silico* analysis reveal that hesperetin exhibits drug-like properties which projects its potential as a therapeutic option for CHIKV infection (Oo et al., 2016).

#### Harringtonine

In an immunofluorescence-based screening platform, harringtonine, a cephalotaxi alkaloid, inhibits early stage of CHIKV replication cycle which occurs after viral entry into cells (Kaur et al., 2013).

#### Aplysiatoxin

Aplysiatoxin related compound extracted from marine cyanobacteria *Trichodesmium erythraeum* has also been found to exhibit dose dependent inhibition of CHIKV (Gupta et al., 2014).

## Traditional Indian Medical System to Combat CHIKV

Dilip et al. (2010) compared the traditional system of medicines in India like Ayurveda, Unani, Homeopathy. Homeopathy drugs such as, Eupatorium, Pyroginum, Nux Vomica, Gelsemium, Arnica, and Belladonna were used for the treatment and prevention of CHIKV and were found to provide 70–80% relief in symptoms within 5 days. Similarly, Ayurvedic method of medication with Vanatulasi patra, Beruenna oil, Vilwadi gulika, Vettumaran gulika, and Sudarshan tablets, etc., showed promising effects in the treatment and prevention of CHIKV. Hence, it was concluded from the study that all these methods of treatment are equally important for the management of chikungunya (Dilip et al., 2010). Since these studies were performed on a limited scale, hence further investigations are required to evaluate the effectiveness of the aforementioned traditional medicines. A homeopathy therapy in acute phase and post-CHIKV chronic arthritis reported 90% recovery of chronic arthritis patients after an average time of 32.5 days. Initial doses of the homeopathic medication was three times a day gradually being reduced to two and then one with improvement (Wadhwani, 2013). Although, the study was promising yet authors did acknowledge that it had certain experimental limitations. Hence, a revised study overcoming the constraints could indubitably establish its efficacy. The extracts and compounds isolated from Tectona grandis were found to be promising drug candidates to both the Asian and ECSA strains of CHIKV (Sangeetha et al., 2017; Martins et al., 2020). Jain et al. (2018b) tried combinations of herbal formulations, practiced by herbal medicine practitioners according to AYUSH, Govt. of India guidelines, for CHIKV treatment. These herbal formulations like Giloy (*Tinospora cordifolia*), Spirulina (*Spirulina platensis*), Ashwagandha (*Withania somnifera*) and Sonth (*Zingiber officinale*) are effective against CHIKV and 100% recovery after 10 days of treatment was observed.

## Conclusion and Research Gap

Chikungunya outbreak is a huge public health concern in India. Twenty-four Indian states and six union territories are endemic for CHIKV. Despite causing acute and self-limiting disease with very low mortality, CHIKF is a challenge to the human population because of the painful course of illness and long-term sequelae which affect the quality of life negatively. Therefore, it is important to ensure its prevention and control measures. Year wise increase of the CHIKV epidemics in India is an alarming sign that prompted us to focus on the understanding about virus biology, virus-vector/virus-host interaction, vector distribution and transmission, strain mutation and circulation. Developing extensive clinical and laboratory diagnostic protocols to delineate CHIKV infection from other flaviviruses like dengue, zika and other alphaviruses such as o’nyong’nyong virus, Sindbis virus and malaria for effective diagnosis is the need of the hour. Continuous surveillance has already been advocated by several studies in India and it should be supported for the apparent reasons. This is very important for early detection of adaptive changes in the viral genome, and prompt risk assessment that would encourage implementation of intervention and control measures to moderate the impact of CHIKV outbreaks in India in the future. A number of novel mutations have been reported in Indian studies that might have effect with respect to adaptability of the virus, spread and disease severity. More research is required to describe the significance of many of the observed mutations considering the fact that the E1-A226V is absent in many Indian epidemics. Till now no licensed vaccine is available, however; several potential vaccine candidates have been identified. Even though many drugs have shown effective CHIKV antiviral activities, studies on clinical efficacy to prove the same is still required. Apart from the conventional drug research, India is also focusing on the research on the traditional system of medicines in our country, like Ayurveda, Unani and Homeopathy which is showing promising results. CHIKV eradication is not only a matter of drugs and vaccines but responsible preventive measures are also to be taken. Until such strategies are made available, simple measures like use of mosquito repellents, expulsion of standing water where mosquitos can lay eggs and minimization of skin surface exposed to mosquito bites are the only measures to prevent this disease.

In the wake of the resurgent viral infectious disease including Zika, Nipah, and the covid-19 pandemic there has been tremendous advancement for existing technologies and disease diagnostics such as the CRISPR system, surveillance; therapeutics such as the use of convalescent plasma; and vaccine development such as the mRNA vaccine. The massive employment of bioinformatics and artificial intelligence tools in tracking infectious pathogen mutations and its implication on disease severity and transmission efficiency can also be used in studies of other viral infections like CHIKV. This suggest that developing a bioinformatic infrastructure for an infectious pathogen can be very helpful to track and make a treatment and prevention strategy to control disease spread. The quest for CHIKV vaccines the mRNA vaccine technology offers new hope.

## Members of the Translational Research Consortia (TRC) for Chikungunya Virus in India

**Anitha Jagadesh**, **Anup Jayaram**, **Naren Babu**, **Piya Paul Mudgal**, **Robin Sudandiradas**, **Shahin Sheik**, and **Ujwal Shetty**: Manipal Institute of Virology, Manipal Academy of Higher Education, Manipal, Udupi, Karnataka, India; **Dileep Kumar Verma**, **Shakuntala Mahilkar**, Sujatha Sunil*, and **Sylvester Agha Ibemgbo**: Vector-Borne Diseases Group, International Centre for Genetic Engineering and Biotechnology (ICGEB), New Delhi, India. **Prabhudutta Mamidi**, **Sharad Singh**, Soma Chattopadhyay* and **Sweta Smita Pani**: Infectious Disease Biology, Institute of Life Sciences, (Autonomous Institute of Department of Biotechnology, Government of India), Nalco Square, Bhubaneswar, India. **Bijayanthimala Mishra**: Department of microbiology, All India Institute of Medical Sciences, (AIIMS), Bhubaneshwar, India; **R. K. Ratho**: Department of Virology, Post-Graduate Institute of Medical Education and Research, Chandigarh, India; **Jayanthi S. Shastri** and **Sachee Agarwal**: Department of Microbiology, TNMC and BYL, Nair Charitable Hospital, Mumbai, Maharashtra, India.

## Author Contributions

SSu, SC, AJg, PPM, BM, RR, JS, and SA took part in Conceptualization and reviewing the manuscript. AJy, NB, RS, SSh, US, DK, SI, SM, PM, SSi, and SP drafting, revising, and preparation of the manuscript. SSu, SC, SM, and SI finalized and prepared the manuscript for submission. Further revisions according to the reviewer comments were made by SSu and SM and finalized by SSu.

## Conflict of Interest

The authors declare that the research was conducted in the absence of any commercial or financial relationships that could be construed as a potential conflict of interest.
