# Supplementary material for: Current Status of Chikungunya in India
Source: Front Microbiol. 2021 Jun 24;12:695173. doi: 10.3389/fmicb.2021.695173 (PMC8274422; doi:10.3389/fmicb.2021.695173)
Supplement: Supplementary file 1 [file Data_Sheet_1.PDF]

SUPPLEMENTARY INFORMATION

Supplementary Table S1: State-wise distribution of laboratory confirmed Chikungunya cases in India (2006- 2019)

—	Indian States / UTs	Years								
		2006	2008	2009	2012	2015	2016	2017	2018	2019
1	Andhra Pradesh	248	1	117	316	83	147	108	79	88
2	Arunachal Pradesh	0	0	0	0	6	8	0	1	55
3	Assam	0	0	0	0	0	40	41	3	0
4	Bihar	0	0	0	0	1	566	1251	156	594
5	Goa	2	21	685	0	32	49	48	77	366
6	Gujarat	225	53	169	221	42	847	1363	1290	669
7	Haryana	0	20	0	0	1	1970	6	3	0
8	Jammu & Kashmir (UT)	0	0	0	0	0	1	0	1	0
9	Jharkhand	0	0	0	0	0	14	17	851	169
10	Karnataka	298	1008	3164	15	2099	1528	3511	2546	3664
11	Kerala	43	492	711	178	152	129	74	77	109
12	Madhya Pradesh	106	0	5	0	11	862	858	1609	756
13	Meghalaya	0	0	0	0	15	68	45	2	48
14	Mizoram	0	0	0	0	0	0	0	10	0
15	Maharashtra	804	238	443	383	207	2949	1438	1009	1646
16	Manipur	0	0	0	0	0	0	0	0	3
17	Odisha	34	11	2	122	46	15	0	0	21
18	Punjab	0	0	0	0	18	2054	201	25	11
19	Rajasthan	24	3	106	1	7	2215	1612	254	365
20	Sikkim	0	0	0	0	0	5	8	28	95
21	Tamil Nadu	116	0	1053	753	329	86	131	284	623
22	Telangana	0	0	0	0	149	71	58	489	1358
23	Tripura	0	0	0	0	7	70	64	75	125
24	Uttar Pradesh	0	7	0	0	0	2458	103	58	72
25	Uttarakhand	0	0	0	0	0	10	0	7	1
26	West Bengal	21	593	338	445	61	117	577	23	NR
27	Andaman & Nicobar Islands (UT)	0	0	0	0	3	0	17	27	53
28	Chandigarh (UT)	0	0	0	0	1	272	54	4	0
29	Dadra & Nagar Haveli (UT)	0	0	0	0	0	0	0	0	0
30	NCT Of Delhi (UT)	67	14	18	0	64	9793	940	407	520
31	Lakshadweep (UT)	0	0	0	0	0	0	0	NR	NR
32	Puducherry (UT)	9	0	0	0	8	20	23	361	794

UT= Union Territories; NR= Not Reported; Data source: National Vector Borne Disease Control Program (NVBDCP), Directorate General of Health Services (DGHS), Ministry of Health and Family Welfare (MoHFW), Government of India & National Health Profile (NHP), Central Bureau of Health Intelligence (CBHI), DGHS, MoHFW, Govt. of India; 2012 data was taken from Integrated Disease Surveillance Program (IDSP) weekly reports, National Center for Disease Control, DGHS, MoHFW, Govt. of India; Data not available for year 2007, 2010, 2011, 2013, and 2014.

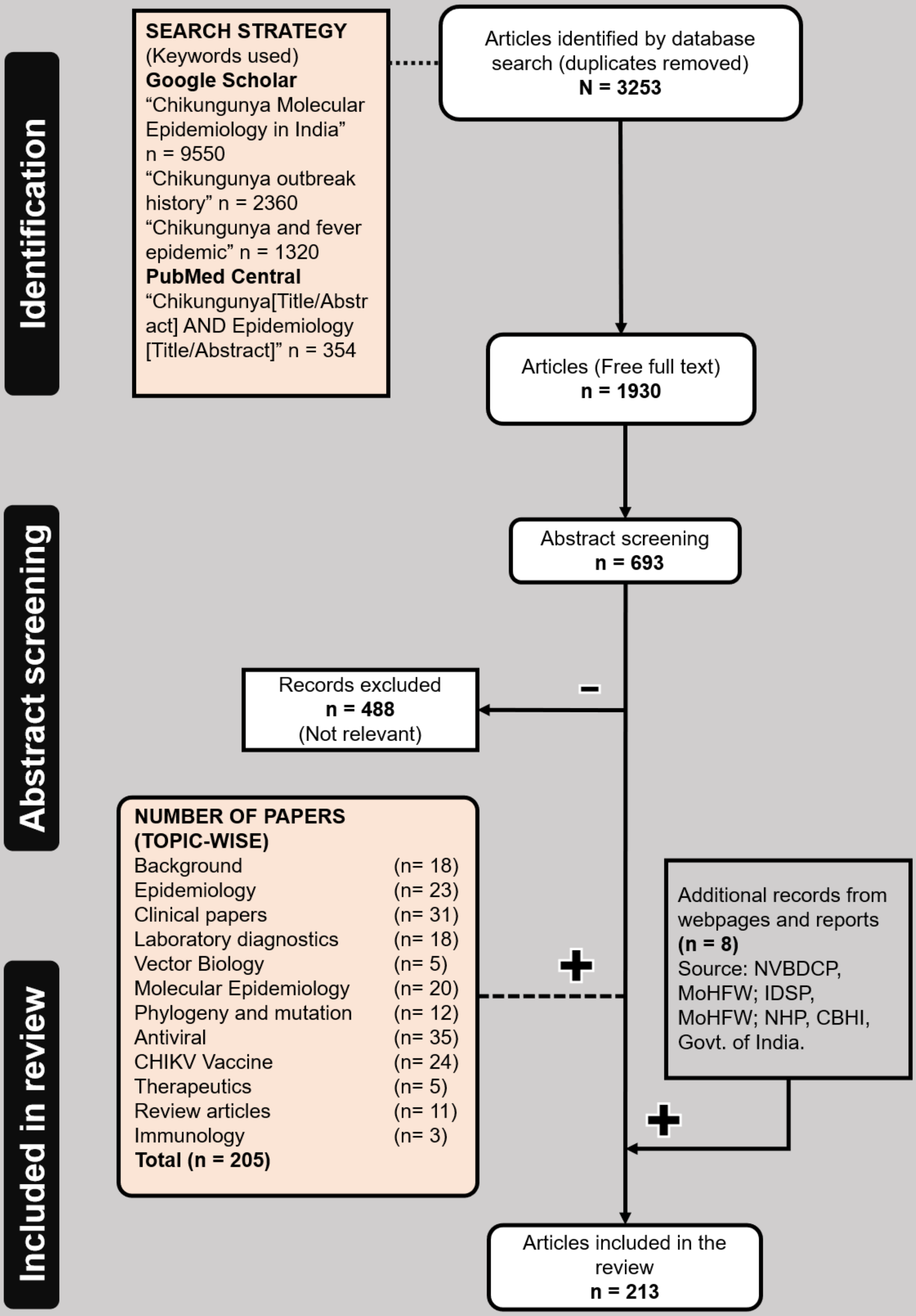

Flowchart showing the methodology used for identification and selection of articles in this review

Supplementary Figure S1. Flowchart showing the methodology used for identification and selection of articles in this review.

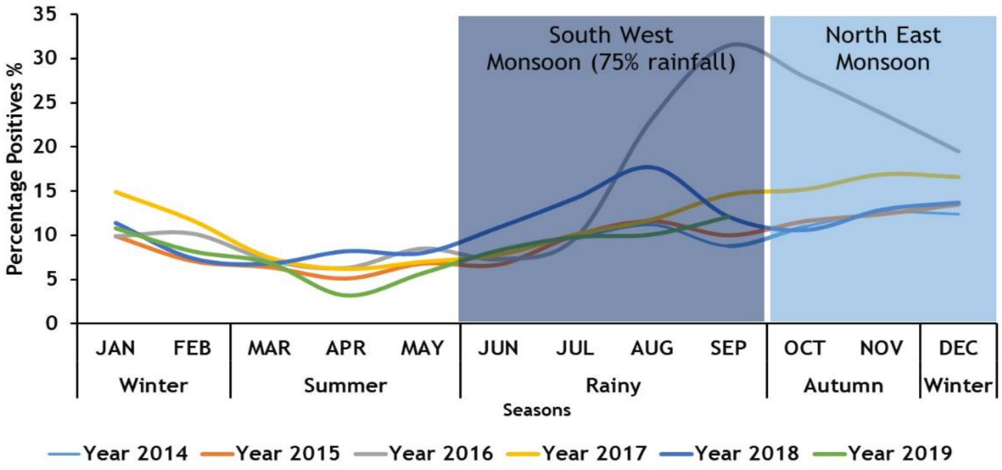

Supplementary Figure S2. Ppercentage positivity of Chikungunya (2014 -19)

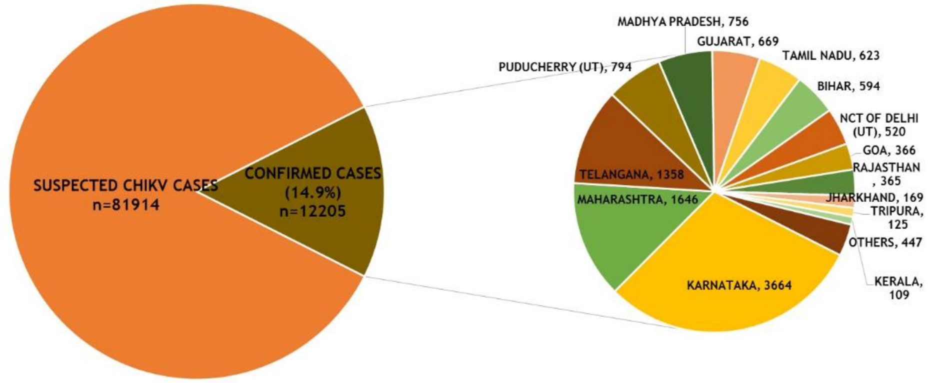

Supplementary Figure S3. State-wise burden of Chikungunya in India – 2019
